# ASFVdb: an integrative resource for genomic and proteomic analyses of African swine fever virus

**DOI:** 10.1093/database/baaa023

**Published:** 2020-04-15

**Authors:** Zhenglin Zhu, Geng Meng

**Affiliations:** 1 School of Life Sciences, Chongqing University, Chongqing, China; 2 Laboratory of Biomedical Research and College of Veterinary Medicine, China Agricultural University, Beijing, China

**Keywords:** African swine fever virus, population genomics, database

## Abstract

The recent outbreaks of African swine fever (ASF) in China and Europe have threatened the swine industry globally. To control the transmission of ASF virus (ASFV), we developed the African swine fever virus database (ASFVdb), an online data visualization and analysis platform for comparative genomics and proteomics. On the basis of known ASFV genes, ASFVdb reannotates the genomes of every strain and newly annotates 5352 possible open reading frames (ORFs) of 45 strains. Moreover, ASFVdb performs a thorough analysis of the population genetics of all the published genomes of ASFV strains and performs functional and structural predictions for all genes. Users can obtain not only basic information for each gene but also its distribution in strains and conserved or high mutation regions, possible subcellular location and topology. In the genome browser, ASFVdb provides a sliding window for results of population genetic analysis, which facilitates genetic and evolutionary analyses at the genomic level. The web interface was constructed based on SWAV 1.0. ASFVdb is freely accessible at http://asfvdb.popgenetics.net.

## Introduction

African swine fever (ASF), caused by ASF virus (ASFV), is a viral disease in domestic or wild swine (1–3). The mortality rate of ASF is nearly 100% for domestic swine infected with virulent strains. The outbreaks in China and central Europe in the summer of 2018 caused important economic losses in the global swine industry. However, efforts to produce effective vaccines or treatments for ASFV are obstructed by the complexity of the virus. ASFV, which presents an icosahedral capsid with a multilayered membrane structure, contains many polypeptides, the function and identities of which are largely unknown. The generated antibodies are not protective (4–8) due to the complexity of the virion and the absence of a neutralizing epitope. Consequently, identification of functional ASFV polypeptides is important for developing effective vaccines.

Due to the above observations, we developed the African swine fever database (ASFVdb), to assist in ASFV research. Different from previous relevant virus databases, such as ViralZone (9) and VBRC (www.4virology.net), ASFVdb is specifically designed for online interactive analysis of ASFV. ASFVdb compares all the published ASFV genomes (10–17), annotates each genome and validates the persistence of each gene in different strains. ASFVdb was developed to facilitate the identification of gene function and identity. In addition to basic annotation, it also provides gene subcellular location and function information, topology predictions, comparative genomic alignments and population genetic analysis results.

## Materials and methods

### Data collection and processing

We downloaded the genome sequences, CDSs, proteins and relevant annotations of 45 sequenced ASFV strains (Table S1) from the NCBI genome database. Among the three different versions (NC_001659.2, U18466.2 and KP055815.1) of Spain_BA71_1971 genomic data, we used the original version, KP055815.1. Of the 45 different ASFV strains with genomic data, only 30 have fully annotated genome, and their gene IDs or names are not unified. To eliminate the inconsistency, we constructed a unified dataset and used the dataset to reannotate the 45 genomes. Specifically, we performed pairwise alignments for all of the annotated protein sequences. The sequences were clustered together with an identity >90% and a coverage >70% according to previous homologous gene identification methods (18, 19). Finally, a list containing 457 clusters was generated. We mapped the representative sequences of these clusters to all 45 genomes. If a representative sequence consisted of multiple mappings with an identity >50% and a coverage >80%, we took the mapping with the highest score and considered the mapped region to be a candidate ORF. To check whether a candidate ORF could express a protein, we predicted its transcription using NCBI ORFfinder and aligned all the possible translations to the queried representative sequence. We took the alignment with the highest score and checked whether its identity and coverage were both higher than 0.5. If they were, we considered ORF expression a possibility; if not, we marked the region as ‘Genetic Remains’. In the database, the genes with NCBI annotations are marked as ‘Annotation from NCBI’, and ORFs identified by us are marked as ‘Newly Annotated’. Finally, we used CD-HIT (20) to perform clustering (requiring an identification >90% and a coverage >70%) and grouped individual ASFV genes into 457 clusters.

To retrieve annotation information for the ASFV gene products from UniProt (21, 22), we BLASTed the protein sequence of individual ASFV genes against the UniProt protein database (22) and chose the hit with the highest score as the best match. If the best match of an individual ASFV gene had an E-value <0.05, we extracted the accession number of the mapped UniProt protein as the UniProt ID of the matched ASFV gene. With the UniProt ID, we obtained external annotations for the ASFV gene, such as the corresponding PubMed ID, EMBL ID, Proteomes ID, Pfam, InterPro ID, GO, KEGG and functional annotations. We also BLASTed all of the ASFV protein sequences against the PDB (23, 24) in the same way and recorded the alignment results. Disappointingly, we obtained only 52 proteins from the PDB that had an E-value <0.05, meaning that most of the ASFV-expressed proteins did not have structural information.

### Subcellular localization and topology prediction

The subcellular location and topology annotations from UniProt covered only some ASFV genes. Thus, we reperformed these predictions for all of the genes. We predicted the subcellular location of the ASFV proteins through MSLVP (25) using ‘one-versus-one’, ‘Second-tier Algorithm’ and a similarity >90%. A total of 457 clusters were grouped into nine subcellular locations. We predicted transmembrane helixes within the protein sequences using TMHMM 2.0 (26). The output images were converted into PNG format to be displayed on the website by ImageMagick (www.imagemagick.org).

### Comparative genomic and population genetic analyses

To trace the evolutionary history of ASFV, we performed whole-genome alignment of all 45 ASFV genomes by Mugsy (27) and built a phylogenetic tree by FastTree 2.1 (28), using the parameter ‘-boot 5000’ to test the likelihood of the generated tree. To obtain a more detailed comparative genomic map of the 45 strains, we used LASTZ (29) to perform genome-genome alignments between pairs of ASFV strains and outputted the results in AXT format. For each ASFV gene, we retrieved the corresponding sequences of other strains from the genome–genome alignment results, realigned these sequences by MUSCLE (30, 31) and obtained the genomic alignment of the CDS region. According to the presence of each gene in different strains, we also retrieved all of the protein counterparts of the gene and performed multiple alignment by MUSCLE. To more clearly display the evolutionary history of each protein, we also drew trees from the multiple alignment data as we did from the genomic data.

We used a window of 200 bp and a step size of 50 bp to slide along the ASFV genome. From the genome–genome alignments described above, we retrieved sequences within the sliding window and used them to calculated Pi, Theta (32) and Tajima’s D (33) using VariScan 2.0 (34, 35). We wrote Perl scripts to call allele frequencies and used SweepFinder2 (36) to calculate the CLR (37, 38) with a step size of 50. We took the medians of the population genetic test statistics in the CDS region as the gene-level values.

### Web interfaces

The web interface of ASFVdb was developed using MysQL + PHP + CodeIgniter (www.codeigniter.com) + jQuery (jquery.com) and based SWAV (39), which is an open source web application for population genetic data visualization and analysis. We used MSAViewer (40) to show the multiple alignments of orthologous CDSs or proteins and PhyloTree (41) to display the phylogenetic trees of genomes or proteins. We made changes to the source code of the web application to fit the development of ASFVdb, such as adding links within the diagram. The search interfaces of the sequence alignments were constructed based on PHP parsing of the results from BLAT (42) and NCBI BLAST (43). The workflows of other search blocks, such as searching by molecular structure or gene name, are mostly SQL query pipelines.

## Results and discussion

### Overview of the genomic data in ASFVdb

**Figure 1:**
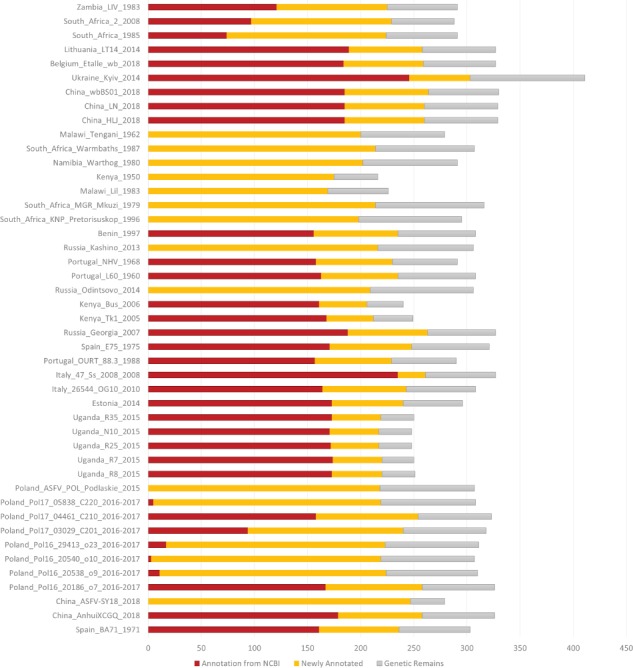
Number of annotated genes in all the ASFV strains. NCBI annotated genes, newly annotated genes and genetic remains are marked in deep red, orange and grey, respectively.

**Figure 2:**
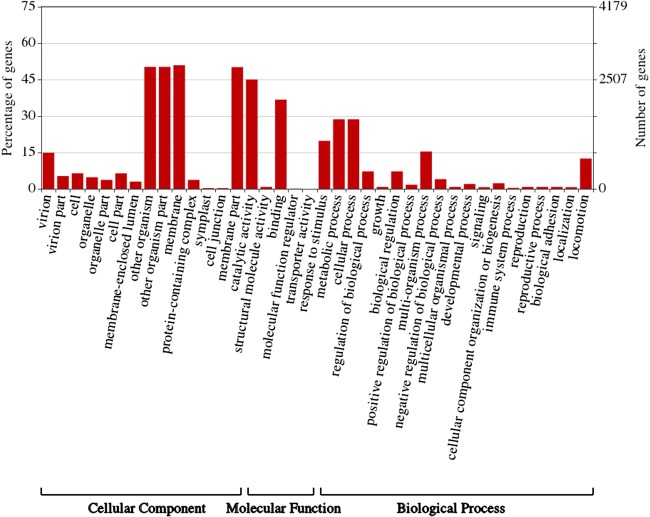
Gene ontology (GO) Enrichment of individual ASFV genes. This figure was created by WEGO (57).

ASFVdb includes a broad range of published ASFV genomic data (Table S1) and analyses. By clustering highly homologous sequences (identity >90%, coverage >70%), ASFVdb groups ASFV individual genes into 457 clusters (Table S2) (for details, see Materials and methods). Among the 457 clusters, 153 have conserved open reading frames (ORFs) in all strains (Table S2). Reannotation of the genomes of the 45 strains predicts 5352 individual genes and 3025 genetic remains (Figure 1). In each strain, there are 169–303 genes. These results do not contradict previous reports of 151–181 genes per strain (17, 44), considering that the pipeline include all possible alternative splices (Figure S1) and the presence of discrepancies between annotations for different strains obtained with different strategies at different times (10–17). We performed subcellular localization analysis of ASFV gene clusters to predict their roles in the infection process. The prediction results indicate that the genes in more than half of the clusters (62%, 282 cases) may encode single-pass or multipass membrane proteins (Table S3). Meanwhile, the genes in 9% of the clusters (43 cases) possibly encode capsid proteins. In Gene Ontology (GO) analysis, the largest numbers of ASFV individual genes corresponds to those that are enriched in the membrane or annotated as having catalytic activity (Figure 2). Moreover, in population genetic analysis, 383 individual genes had a significantly low Tajima’s *D* values (33) and high composite likelihood ratios (CLRs) (37, 38) (rank test, *P* value <0.05). Therefore, it is possible that these genes were recently under positive selection (Table S4). A total of 811 individual genes had a significantly high Tajima’s *D* value (Table S5, rank test, *P* value <0.05) and thus may have been under balancing selection. These results are informative for future ASFV research.

**Figure 3:**
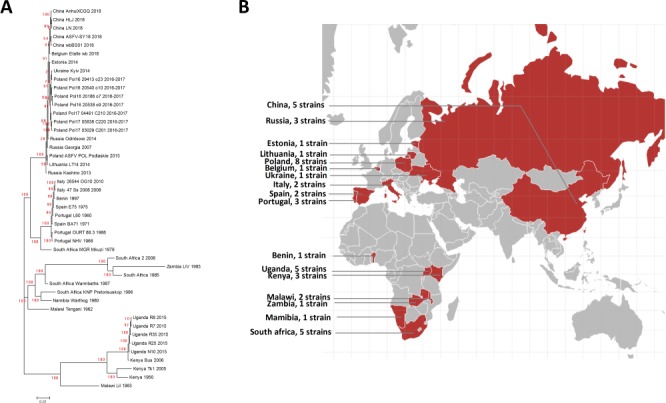
**A**. Phylogenetic tree of the ASFV strains in ASFVdb. The numbers marked in red are the marginal likelihoods of the tree. **B**. The distribution of strains in ASFVdb according to country.

**Figure 4:**
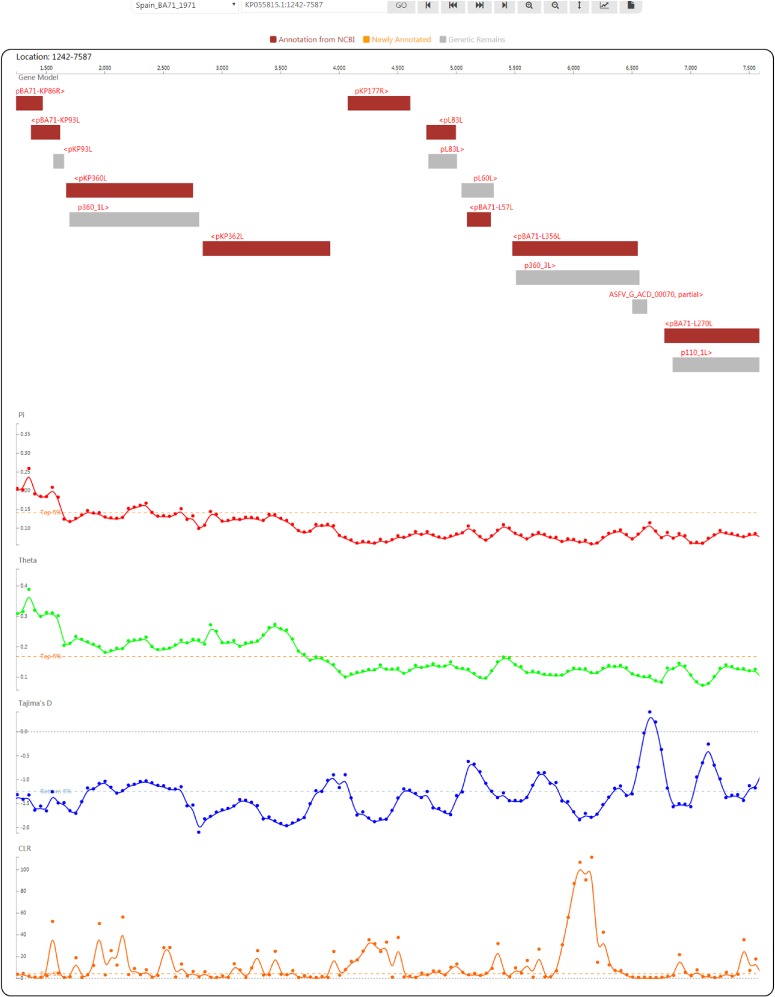
Genome browser view used to display ASFV genes and population genetic test statistics.

### ASFVdb offers a diverse search method

The main search function of ASFVdb, located in the centre of the home page, allows users to search for a gene by its general name, its accession number or a description of its function. We created a gene alias list to make the search results as complete as possible. In the right column of the home page, there is a list of other gene search methods in ASFVdb. First, users can BLAT against one ASFV genome, similar to the workflow in the UCSC genome browser (45), or BLAST against coding sequence (CDS) or protein sequences. Second, users can search for genes in a specific subcellular location. In the results view, we list the topology information and functional annotations of each gene to allow users to conveniently localize the gene in relation to host–virus interaction. Third, users can find genes with a specific function according to GO annotation. ‘Clusters’ shows the representation of the individual ASFV genes in the 45 strains. Users can view a specific gene by clicking an item in the gene list table. Moreover, to more conveniently track a list of genes, ASFVdb provides gene links established by inputting a list of the genomic positions or accession numbers of individual genes. These operations facilitate analysis of a personalized gene list.

### Evolutionary episodes of the ASFV strains in ASFVdb

On the home page, below the main search box, a phylogenetic tree displays the evolutionary history of the 45 published ASFV strains. In contrast to the previous methods used to construct ASFV phylogenetic trees (14, 17), we built the tree by performing multiple alignments at the genomic scale to better understand the patterns of evolution (46). The tree that we constructed (Figure 3) is consistent with the trees in previous publications (17, 44, 47). Our tree reflects transmission events during the spread of ASFV. After it was first identified in Kenya in the 1920s (48), ASFV was subsequently reported in eastern and southern Africa (49). In the middle of the last century, ASFV spread from Africa to Portugal (50), its first stop in Europe. Then, ASFV spread to Malta (1978), Italy (1967, 1980), France (1964, 1967, 1977), Belgium (1985) and the Netherlands (1986) (51). In 2007, ASFV was introduced to Caucasus (52) and began to spread across Russia and Eastern Europe. In 2018, ASFV was found in China (53).

### Gene visualization in ASFVdb

By clicking on the taxon name in the tree or selecting an item in the ‘Gbrowser’ list, users can go to the genome browser page (Figure 4), where gene segments are subsequently arranged along the genome according to the genomic positions of individual genes. Genes annotated from the NCBI GFF file are coloured in deep red to indicate ‘Annotation from NCBI’. Genes annotated by mapping (for details, see the Materials and methods) are coloured in orange to indicate ‘Newly annotated’. ‘Genetic Remains’ are coloured in light grey. The gene name is provided above each gene segment, and ‘>’ or ‘<’ indicates a gene’s transcriptional direction. Four tracks of population genetic test statistics, namely, Pi, Theta (32), Tajima’s D (33) and the CLR (37, 38), are listed following the gene track. To help trace signatures of selection, a top 5% line for the CLR and two 5% lines in ascending and descending order for Pi, Theta and Tajima’s *D* are shown. Using the tool bar at the top, users can move along or zoom in the genome in the browser, set the focus bar to a selected region, export the drawing of one track with gene segments and export the data of one track.

Clicking on a gene segment will lead to the gene’s information page. The full list of annotations includes basic information (strain, gene name, description, location in the genome, GenBank accession number and full name), sequences (CDS and protein sequence), a summary (function, UniProt accession number, related PubMed ID, related EMBL ID, corresponding Proteomes ID, related Pfam ID and correlated InterPro ID), ontologies (GO and Kyoto Encyclopedia of Genes and Genomes, KEGG), subcellular location prediction, topology prediction (transmembrane region prediction), genomic alignment in the CDS region, multiple alignment of orthologues, a gene tree of corresponding NCBI annotated or newly annotated proteins, and orthologous genes in strains. Text that links to internal or external sites is hyperlinked to facilitate viewing and analysis.

### Analysis of CD2v (EP402R) in ASFVdb: a case study

CD2v (EP402R) is an ASFV gene related to haemadsorption (54). This gene plays a key role in viral attenuation and may be involved in the generation of immune protection in the host against ASFV infection (55). By searching for ‘EP402R’ in the strain Spain_BA71_1971, we find that this gene encodes a single-pass membrane protein with a transmembrane region. A total of 2 to 24 amino acids (AA) may correspond to a signal peptide, whereas 206 AA to 228 AA may correspond to the transmembrane domain of this single-pass membrane protein (Figure S2A). In the genome view with the population genetics analysis tracks, we see a Tajima’s *D* valley and a CLR peak (Figure S2B) at the gene’s ORF region, representing signatures of selection. The region from 25AA to 205AA is predicted to be located outside of the virion membrane, with peaks in Pi and Theta, indicating its high diversity among strains. From the information regarding ‘Genomic alignment in the CDS region’ and ‘Orthologues in Strains’, we find that this gene is highly conserved among only 9 of all 45 compared strains (Figure S2C). ASFVdb annotates this protein as being similar (E-value = 0.000874314) to the cell adhesion molecule CD2 (56), the crystal structure of which (Figure S2D, from the Protein Data Bank, PDB) has been determined.

## Conclusion

To assist veterinary scientists and researchers in combating the threat of ASFV outbreaks, we developed a database-based toolbox, ASFVdb. ASFVdb integrates data from NCBI, UniProt (21), ViralZone (9) and a broad range of published articles (9), specializing in ASFV. ASFVdb compares all published ASFV genomes (10–17), summarizes data on the proteins of all known ASFV strains and provides not only basic gene information but also external links, subcellular localization and topological information, comparative genomic alignments, a gene tree and evolutionary analysis results. This information will be helpful and convenient for the further collection of proteomic and biochemical data in ASFV research. Although the 5352 newly annotated possible ORFs have not been validated by experiments, we believe that they are informative for future research. Hopefully, ASFVdb will be useful for further research on ASFV and vaccine design.

## Data availability

All ASFVdb data are publicly and freely accessible at http://asfvdb.popgenetics.net. Feedback on any aspect of the ASFVdb database and discussions of ASFV gene annotations are welcome by email to zhuzl@cqu.edu.cn or mg@cau.edu.cn.

## Author contributions

Z.Z. developed the web interface of the database, collected and compiled the data and performed the analysis. Z.Z. and G.M. wrote the manuscript, conceived the idea and coordinated the project.

## Supplementary Material

Supplementary_Figures_baaa023

Supplemental_Tables_baaa023
