# Supplementary material for: ASFVdb: an integrative resource for genomic and proteomic analyses of African swine fever virus
Source: Database (Oxford). 2020 Apr 15;2020:baaa023. doi: 10.1093/database/baaa023 (PMC7159030; doi:10.1093/database/baaa023)
Supplement: Supplementary_Figures_baaa023 [file supplementary_figures_baaa023.pdf]

Supplementary Figures

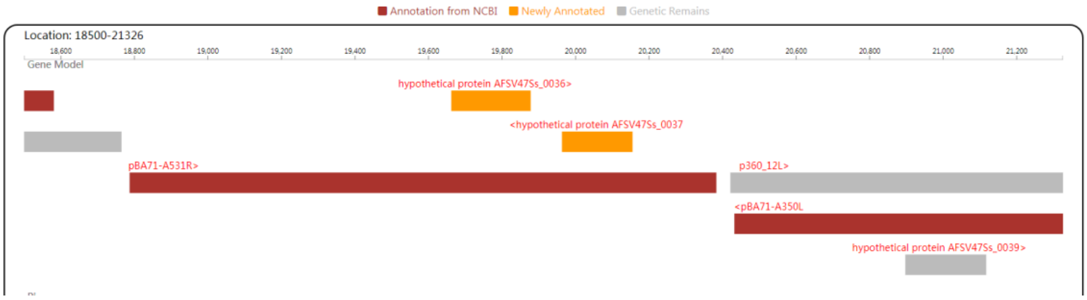

Figure S1. A snapshot displaying the inclusion of alternative splicing cases in ASFVdb. As shown in the figure, ‘pBA71-A531R’, coloured in red, is possibly transcribed in the forms of “hypothetical protein AFSV47Ss_0036” and “hypothetical protein AFSV47Ss_0037”, coloured in orange.

A

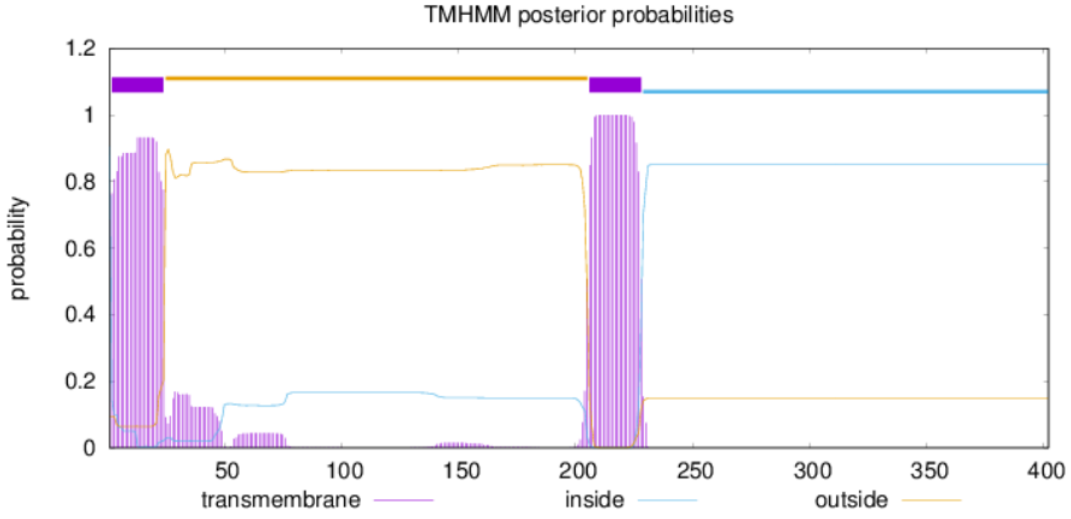

TMHMM posterior probabilities
probability
1.2
1
0.8
0.6
0.4
0.2
0
50
100
150
200
250
300
350
400
transmembrane
inside
outside

B

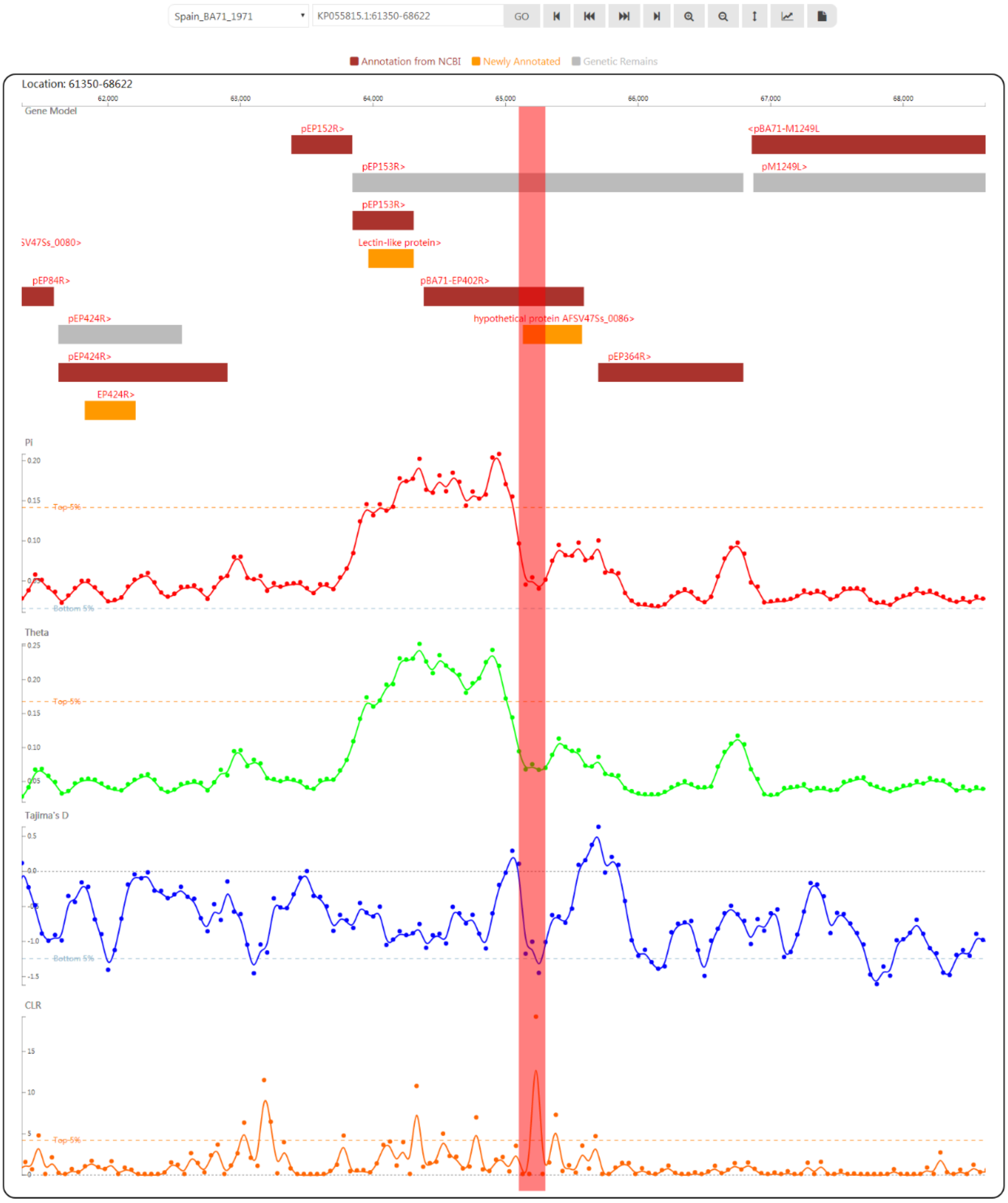

Spain_BA71_1971
KP055815.1:61350-68622
GO
Annotation from NCBI
Newly Annotated
Genetic Remains
Location: 61350-68622
Gene Model
pEP152R>
<pBA71-M1249L
pEP153R>
pM1249L>
pEP153R>
Lectin-like protein>
pEP84R>
pBA71-EP402R>
pEP424R>
hypothetical protein AFSV47Ss_0086>
pEP424R>
pEP364R>
EP424R>
Pi
Top 5%
Bottom 5%
Theta
Tajima's D
CLR

C

Orthologous in Strains

Strain	Availability	Status	Gene
Spain_BA71_1971	✔	Protein	AKO62740.1
China_AnhuiXCGQ_2018	✖		
China_ASFV-SY18_2018	✖		
Poland_Pol16_20186_o7_2016-2017	✖		
Poland_Pol16_20538_o9_2016-2017	✖		
Poland_Pol16_20540_o10_2016-2017	✖		
Poland_Pol16_29413_o23_2016-2017	✖		
Poland_Pol17_03029_C201_2016-2017	✖		
Poland_Pol17_04461_C210_2016-2017	✖		
Poland_Pol17_05838_C220_2016-2017	✖		
Poland_ASFV_POL_Podlaskie_2015	✖		
Uganda_R8_2015	✖		
Uganda_R7_2015	✖		
Uganda_R25_2015	✖		
Uganda_N10_2015	✖		
Uganda_R35_2015	✖		
Estonia_2014	✖		
Italy_26544_OG10_2010	✔	Protein	AJZ77076.1
Italy_47_Ss_2008_2008	✔	Protein	AOO54390.1
Portugal_OURT_88.3_1988	✔	Protein	CAN10406.1
Spain_E75_1975	✔	Protein	CBH29159.1
Russia_Georgia_2007	✖		
Kenya_Tk1_2005	✖		
Kenya_Bus_2006	✖		
Russia_Odintsovo_2014	✖		
Portugal_L60_1960	✖		
Portugal_NHV_1968	✔	Protein	AIY22408.1
Russia_Kashino_2013	✖		
Benin_1997	✔	Protein<br>Protein	CAN10158.1<br>CAN10158.1
South_Africa_KNP_Pretorisuskop_1996	✖		
South_Africa_MGR_Mkuzi_1979	✔	Protein	AIY22249.1
Malawi_Lil_1983	✖		
Kenya_1950	✖		
Namibia_Warthog_1980	✔	Protein	QGM12720.1
South_Africa_Warmbaths_1987	✖		
Malawi_Tengani_1962	✖		
China_HLJ_2018	✖		
China_LN_2018	✖		
China_wbBS01_2018	✖		
Ukraine_Kyiv_2014	✖		
Belgium_Etalle_wb_2018	✖		
Lithuania_LT14_2014	✖		
South_Africa_1985	✖		
South_Africa_2_2008	✖		
Zambia_LIV_1983	✖		

D

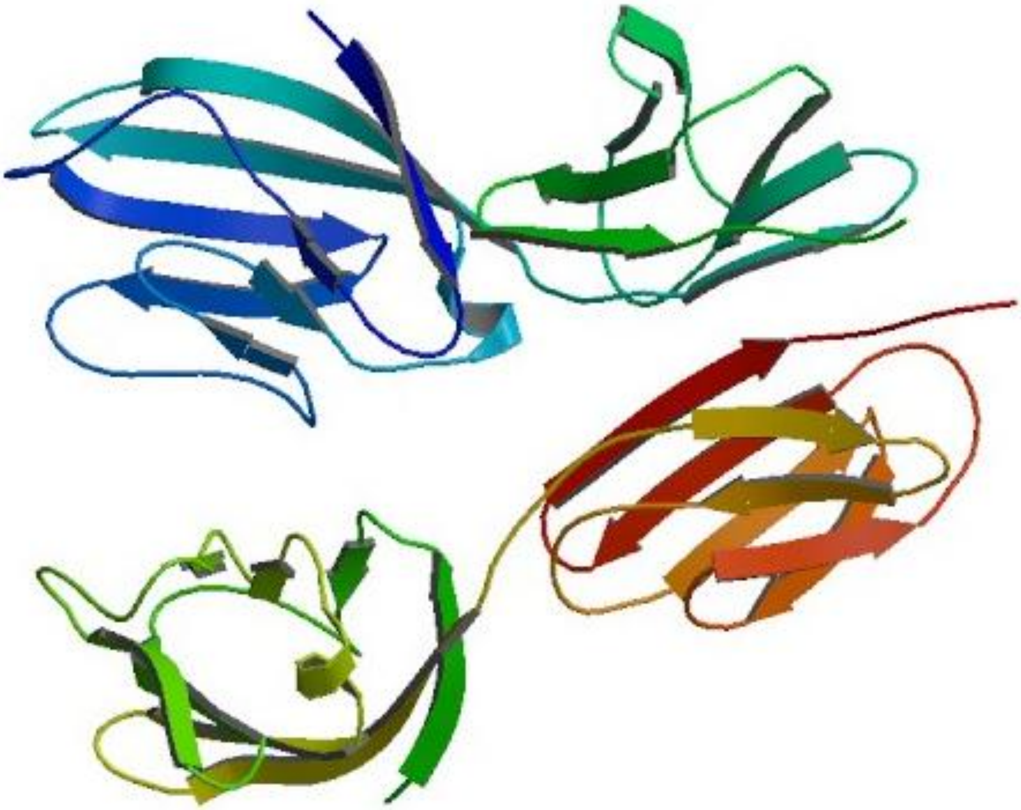

Figure S2. Analysis results of EP402R in ASFVdb. A shows the transmembrane prediction of EP402R in Spain_BA71_1971. B shows the population test statistic results for Spain_BA71_1971, where the red bar marks the region with a Tajima's D valley and a CLR peak in EP402R. C shows the representation of EP402R in different strains. D shows the 3D structure of the cell adhesion molecule CD2 from the PDB, which is similar to EP402R (E-value<0.05).
